# The impact of historical redlining policies on community composition and the COVID-19 pandemic in Boston

**DOI:** 10.1371/journal.pone.0324020

**Published:** 2025-06-18

**Authors:** Nicole B. Alkhouri, Victoria Fisher, Isaacson Michel, Caitlin O’Connor, Nadia N. Abuelezam

**Affiliations:** 1 Boston College William F. Connell School of Nursing, Chestnut Hill, Massachusetts, United States of America; 2 Michigan State University College of Human Medicine, East Lansing, Michigan, United States of America; Georgetown University Medical Center, UNITED STATES OF AMERICA

## Abstract

The COVID-19 pandemic has accentuated racial and socioeconomic disparities that have existed in the U.S. because of structural racism. We aimed to understand how the legacy of historical redlining policies was associated with COVID-19 incidence in Boston ZIP code tabulation areas (ZCTAs) from March 2020 to December 2022. Data were extracted from the Institute for Social Research at the University of Michigan, the Boston Public Health Commission, the Boston Planning & Development Agency, and the American Community Survey. We ran a generalized linear model accounting for time to explore the association between historical redlining, characterized as Homeowners’ Loan Corporation (HOLC) grade, and monthly COVID-19 incidence rate. Models were adjusted for the proportion of the ZCTA population identified as non-White, were older than 75, homeowners, and foreign-born. We also accounted for the median household value in each ZCTA. We found no significant association between HOLC grade and monthly COVID-19 incidence rate in Boston (IRR: 0.95; 95% CI: 0.80, 1.12). Downstream effects of redlining, such as gentrification and higher median home value, were associated with a higher monthly COVID-19 incidence (IRR: 1.12; 95% CI: 1.01, 1.25). These results highlight the unique ways Boston’s historical racist policies have manifested themselves in health outcomes today. Place-based context and history is important when examining redlining in public health research.

## Introduction

COVID-19 has been ravaging communities across the United States (U.S.) since March of 2020. The COVID-19 pandemic has brought attention to racial and socioeconomic disparities that have existed in the U.S. that can largely be attributed to structural racism [1]. Structural racism has been linked to a number of health issues including preterm birth [2], mental health outcomes [3,4], and COVID-19 in communities of color [5]. Current disparities in health are a product of historical injustices and policies that impact both the living environment and health outcomes [1,6].

One historical policy with long term implications for health and wellness is redlining. Redlining policies segregated communities on racial and ethnic lines [1,7]. In the 1930s, cities across the U.S. were demarcated and distinguished based on potential investment value by the Homeowners’ Loan Corporation (HOLC) in collaboration with the Federal Housing Administration (FHA) [8,9]. The HOLC created maps that distinguished neighborhoods based on a predetermined rating system that influenced different groups’ ability to obtain home loans. Neighborhoods were given one of four letter grades: ‘A’ (Best), ‘B’ (Desirable), ‘C’ (Declining), and ‘D’ (Hazardous) [1,7]. Neighborhoods awarded the grades ‘A’ or ‘B’ were predominately White wealthy neighborhoods that easily received home loans, and those graded ‘C’ or ‘D’ were occupied by the working class, European immigrants, Mexican, and Black/African American communities [1,7]. Redlining had a negative impact on neighborhood development [8,10]. Reduction in credit access led to lower demands for housing, which directly led to a decrease in housing investment; homeowners were left owing more than the house’s market value [11,12]. This eventually led to poor upkeep for properties as their value diminished [8,13–15]. Active divestment in neighborhoods deemed “hazardous” or “declining” led to increased rates of home vacancies and their dilapidation [6]. A neighborhood’s rating impacted long-term investment into the community. The passing of the Fair Housing Act of 1968 called for the legal end of housing discrimination. While redlining policies have been outlawed, their consequences are still being seen decades later [10,16].

One significant implication of redlining was demographic change in neighborhood composition influenced by local and federal practices. The historical patterns in Boston, Massachusetts (MA) exemplify these changes. A small proportion of Black/African American residents in any Boston neighborhood brought the HOLC rating down, despite a quality environment [17]. Roxbury, a neighborhood in Boston, was given a “hazardous” rating due to an “infiltration of Negroes [17,18].” European immigrant groups, including Irish, Jewish, and Italian residents, were also among those affected by redlining practices in Boston [19]. After the Fair Housing Act of 1968 passed, the Boston Banks Urban Renewal Group (B-BURG) was formed to provide Black Bostonians loans for home ownership and to correct past injustices [17,20]. Black/African American residents were limited to purchasing homes exclusively in areas located in Roxbury, Dorchester, and Mattapan [20]. Purchases outside of these designated areas were denied [21]. The influx of Black/African American residents, into what formerly were predominantly Jewish communities, caused the Jewish residents to leave [17]. This process, called ‘blockbusting,’ involved real estate brokers scaring off residents to quickly sell their homes [21]. As decades passed, these neighborhoods became predominantly Black/African American communities [22,23]. This program ultimately failed to alleviate housing discrimination [20,21]. Homes purchased through B-BURG by Black/African Americans residents were poorly maintained and the residents lacked the resources to improve their housing conditions [21]. The inability to take care of their homes and to build equity led to foreclosures and abandonment [21].

Housing is a well-established social determinant of health. Poor living conditions translate to increased vulnerability to infectious diseases and poor health outcomes. A study from Louisiana linked neighborhoods most deprived of resources to increased risk of contracting COVID-19 [24,25]. Adverse housing factors, such as crowding, poor ventilation, and deprivation have been shown to have a negative impact on health [10,26]. Exposure to mold, dampness, pest, and toxic substances was associated with respiratory issues, lung cancer, and chronic illness [27–33]. Redlined communities were more susceptible to chronic illness due to a lack of investment in housing infrastructure and maintenance [10,33]. A nationwide study across U.S. counties observed an increase in COVID-19 incidence and mortality associated with the percentage of households with poor housing conditions [34]. Cumulatively, these studies suggest that housing influences infectious disease risk.

Prior studies have focused on the impact of redlining policies on chronic disease incidence in New York [2,35], while others have examined the association between redlining and COVID-19 outcomes in both New York City and California [6,36,37]. To our knowledge, little or no prior work has examined how the legacy of redlining has impacted infectious disease outcomes in Boston, MA [6,26]. We aim to understand how historical redlining policies were associated with COVID-19 incidence in Boston from March 2020 through December 2022.

## Methods

### Data

Redlining data were extracted at the census tract level from the Institute for Social Research at the University of Michigan [38]. COVID-19 incidence rates were extracted from the Boston Public Health Commission’s COVID-19 Dashboard [39]. Boston’s demographic data was obtained from the 2020 Decennial data and the 2016–2020 American Community Survey via the Boston Planning & Development Agency [22,40,41].

### Outcome

The primary outcome for this study was the COVID-19 incidence rate, defined as the number of COVID-19 cases per 10,000 residents [39]. We calculated this by dividing the monthly COVID-19 cases per ZIP code tabulation area (ZCTA) by the annual population of ZCTA. This analysis examined monthly incidence rates from March 2020 through December 2022 [39].

### Exposures

The primary exposure was the numerical indicator of the HOLC grade of a ZCTA [38]. Grades were converted to a continuous numerical range of 1–4, averaged based on the proportion of the ZCTA corresponding to each grade. A higher numerical score indicates greater overall redlining, such that A = 1, B = 2, C = 3, and D = 4, etc. The HOLC scores are given at the Census Tract level and were aggregated up to ZCTA using the Department of Housing and Urban Development (HUD) 2010–2020 census tract to ZCTA crosswalk files. Only ZCTAs with historical redlining and COVID-19 data were included in the analysis (N = 25). Two ZCTAs did not have COVID-19 cases and three had no historic redlining data (S1 Fig in S1 File).

### Covariates

The final model was adjusted for a variety of covariates based on a priori knowledge, and data availability. We adjusted for time, the proportion of the ZCTA population older than 75, the proportion of the ZCTA population identifying as foreign-born, the proportion of the ZCTA population identifying as non-White, standardized median household values, and the proportion of the ZCTA population that owned their dwelling unit.

### Statistical analysis

After excluding missing data in COVID-19 cases, HOLC grades, and covariates across the entire study period, 25 ZCTAs were included in the final analysis across 34 months, totaling 850 observations. The incidence rate was log-transformed to obtain a normal distribution. Generalized linear models (glm) with gaussian family were conducted to account for ZCTA repeated measures.

The statistical analysis was performed in R 4.2.1. The code used for this research is available on GitHub {https://github.com/victoriapfisher/Redlining_PLOSOne.git}.

## Results

### Descriptive statistics

On average, 54% of all ZCTA residents identified as White (95% CI: 44%, 64%), and 46% identified as non-White (95% CI: 36%, 56%) (Table 1). Of those who identified as non-White, 17% identified as Hispanic (95% CI: 12%, 22%), 20% identified as non-Hispanic Black (95% CI: 11%, 29%), and 11% identified as Asian/Pacific Islander (95% CI: 7%, 15%). For ZCTAs included in this analysis, mean historical redlining score was 3.4 (95% CI: 3.2, 3.5) and mean COVID-19 incidence rate per 10,000 people was 105.3 (95% CI: 85.2, 125.5).

**Table 1:** Average demographics across Boston ZIP code tabulation areas (ZCTAs) (N = 25), 2020–2022.

Demographics	Mean (95% CI)
COVID-19 incidence rate (10k)	105.3 (85.2, 125.5)
Historical redlining indicator score	3.4 (3.2, 3.5)
75 years and older (%)	4.7 (4.0, 5.4)
Median household value ($)	736106 (614916, 857296)
Less than a bachelor’s degree (%)*	79.1 (75.2, 82.9)
Foreign born (%)	26.7 (22.9, 30.5)
Non-Hispanic White	54.2 (44.4, 64.1)
Non-White population (%)	45.8 (35.9, 55.6)
Asian/Pacific Islander	11.1 (6.9, 15.4)
Non-Hispanic Black	19.8 (10.9, 28.8)
Hispanic	17.0 (12.4, 21.6)

### Regression results

There was no significant association between historical redlining of Boston ZCTAs and COVID-19 incidence rate after adjusting for all covariates (IRR: 0.95; 95% CI: 0.80, 1.12) (Table 2). Advanced age (75 years and older) was significantly associated with a lower COVID-19 incidence rate (IRR: 0.88; 95% CI: 0.83, 0.93). Additionally, an increase of one standard deviation in ZCTA median household value corresponded to a 1.12 times higher COVID-19 incidence rate (95% CI: 1.01, 1.25). Similarly, ZCTAs with a higher proportion of homeowners, foreign-born residents, and non-White populations experienced 1.02 (95% CI: 1.00, 1.04), 1.01 (95% CI: 1.00, 1.03), and 1.01 (95% CI: 1.00, 1.01) times the COVID-19 incidence rates, respectively, compared to the mean.

**Table 2:** Generalized Linear Model (GLM) regression results.

Characteristics	IRR (95% CI)
Historical redlining indicator	0.95 (0.80, 1.12)
Time (months)	1.03 (1.02, 1.03)
75 years and older	0.88 (0.83, 0.93)
Median household value	1.12 (1.01, 1.25)
Owner occupied	1.02 (1.00, 1.04)
Foreign- born %	1.01 (1.00, 1.03)
Non-White population (%)	1.01 (1.00, 1.01)

This table shows the association between historical redlining (a continuous indicator that ranges from 1 to 4) and COVID-19 incidence rate per 10,000 residents adjusting for the proportion of the ZCTA population that is 75 years and older, foreign-born, dwelling owners, and non-White. Models were additionally adjusted for median household value and time.

## Discussion

We aimed to examine how historical redlining in Boston was associated with COVID-19 incidence from 2020 to 2022. We found no significant association between redlining and COVID-19 incidence in Boston. However, our results indicated that ZCTAs with higher median household income and a higher proportion of dwelling owners, foreign-born individuals, and non-White individuals had higher COVID-19 incidence.

One facet of the legacy of historical redlining policies manifested as poor housing and worse living conditions, which previous studies reported were associated with infectious disease outcomes [34,42–44]. As such, we used median household value as a proxy for housing conditions as higher median values often indicate neighborhood living conditions [45]. However, for the data included in this analysis, median household value in 2020 and 2021 was significantly associated with higher redlining scores, or worse original grades [S1 Table in S1 File]. In Boston, disinvestment allowed for developers and opportunists looking to purchase units at low cost in Boston’s historic neighborhoods and redevelop, or gentrify, into higher value properties [46]. That our study detected no significant association between historical redlining and COVID-19 outcomes is on par with Boston’s development history in the years since redlining was outlawed. Studies in Massachusetts have hypothesized that rising house prices might be associated with displacement pressure for low-income families, resulting in higher COVID-19 transmission in higher median home value areas as low-income residents took up more jobs that required them to work outside the home [46–48].

From a historical perspective, the redlined neighborhoods of Boston were primarily home to European immigrants in the early 20th century—these include South Boston, the North End, and the South End, all areas with high home value today. Introduced in the 1930s, redlining and other racist urban development practices primarily (often explicitly) targeted Black and Brown populations, which comprised less than 2% of Boston’s population at the time [49]. Although White European immigrants were not immune to violence and racism, including redlining, historical records suggest they were targeted by acts of violence at the same rate as White Americans [50]. Today, the historically redlined neighborhoods of Boston continue to be home predominantly to descendants of those White and White-passing immigrant groups–Italian, Irish, and Central European. A consistent increase in Black and Brown residents in Boston did not start until the 1980s and 90s [51,52], by which time redlining was outlawed. As a result, Black and Brown Bostonians experienced different residential contexts than those who may have moved to places such as Detroit, Chicago, and other industrial northern cities during the Great Migration. For example, Mehdipanah et al. found that historically redlined neighborhoods in Detroit, which were and continue to be home to predominantly Black residents, were associated with worse access to critical determinants of health today [53]. This is not to say that non-White Bostonians have greater access to health resources than Black and Brown residents living in multigenerational redlined areas; rather, non-White Bostonians are less likely to live in formerly redlined parts of the city compared to other redlined cities. Indeed, our results suggest that non-White residents were at greater risk for COVID-19 infection, though redlining is unlikely the defining mechanism for this result. Our work adds nuance to the ways in which redlining should be used as an exposure in public health research [54], acknowledging the importance of history and place-based context in infectious disease epidemiology [55].

Through rapid gentrification, historical redlining has impacted the racial composition of neighborhoods in Boston [56]. Our findings were consistent with prior literature linking high non-White community composition with increased COVID-19 risk and incidence [25,53]. A New York City study found that predominantly Black/African American and Hispanic areas had higher COVID-19 infection rates [6]. In Boston, neighborhoods with higher Hispanic populations had 25% higher COVID-19 incidence [57]. At the beginning of the pandemic, infections among Black Bostonians accounted for 40% of the COVID-19 cases [58]. This disproportionate impact can be attributed to factors such as limited access to healthcare, food insecurity, lack of clean water, overcrowded living conditions, and the types of jobs these communities are more likely to hold [59].

Our study had several limitations. Boston is home to a large population of college students. We hypothesize that Boston’s dynamic, transient population may have influenced the concentration of COVID-19 cases, particularly around campuses. Many of the campuses and student housing are located in Boston’s historically redlined communities [60]. Consequently, this could have obscured patterns of health disparities for long-term Boston residents. Additionally, the demographic data we obtained from ACS was annual compared to our outcome data which was monthly. This temporal mismatch could have introduced discrepancies in our analysis. Lastly, the redlining data we utilized was available on the census tract level, which required us to crosswalk the data to ZCTAs. Both ZCTAs and tracts may have undergone significant changes since HOLC grading was implemented, potentially leading to inaccurate description of geographic areas impacted by redlining [7].

Our study adds to a growing body of literature that explores the link between historical redlining and contemporary health disparities. Our findings indicated that downstream effects of redlining, such as gentrification and higher median household value, were associated with higher COVID-19 incidence rates, illustrating the variety of consequences racist historical policies can have on infectious diseases. Future work in other urban contexts is necessary to expand our understanding of how structural factors impact health outcomes beyond COVID-19.

## Supporting information

S1 File Supplementary materials for “The impact of historical redlining policies on community composition and the COVID-19 pandemic in Boston.” This supplement contains an additional table with linear regression results and a redlining map of Historical Redlining Grades.
(DOCX)

## References

[1] Richardson J, Mitchell BC, Meier HCS, Lynch E. The lasting impact of historic “Redlining” on neighborhood health: Higher prevalence of COVID-19 risk factors. National Community Reinvestment Coalition [Internet]; 10 Sep 2020 [cited 16 Sep 2024]. Available from: https://ncrc.org/holc-health/

[2] Krieger N, Van Wye G, Huynh M, Waterman PD, Maduro G, Li W, et al. Structural racism, historical redlining, and risk of preterm birth in New York City, 2013-2017. Am J Public Health. 2020;110(7):1046–53. doi: 10.2105/AJPH.2020.305656 32437270 PMC7287548

[3] Bailey ZD, Feldman JM, Bassett MT. How structural racism works - racist policies as a root cause of U.S. racial health inequities. N Engl J Med. 2021;384(8):768–73. doi: 10.1056/NEJMms2025396 33326717 PMC11393777

[4] Williams DR, Lawrence JA, Davis BA. Racism and Health: Evidence and Needed Research. Annu Rev Public Health. 2019;40:105–25. doi: 10.1146/annurev-publhealth-040218-043750 30601726 PMC6532402

[5] Zalla LC, Martin CL, Edwards JK, Gartner DR, Noppert GA. A geography of risk: structural racism and coronavirus disease 2019 mortality in the United States. Am J Epidemiol. 2021;190(8):1439–46. doi: 10.1093/aje/kwab059 33710272 PMC7989642

[6] Li M, Yuan F. Historical redlining and resident exposure to COVID-19: a study of New York City. Race Soc Probl. 2022;14(2):85–100. doi: 10.1007/s12552-021-09338-z 34178163 PMC8212581

[7] Meier HCS, Mitchell BC. Tracing the Legacy of Redlining: A New Method for Tracking the Origins of Housing Segregation NCRC. National Community Reinvestment Coalition [Internet]; 24 Feb 2022 [cited 16 Sep 2024]. Available from: https://ncrc.org/redlining-score/

[8] Aaronson D, Hartley D, Mazumder B. The effects of the 1930s HOLC “Redlining” Maps. Am Econ J. 2021;13(4):355–92. doi: 10.1257/pol.20190414

[9] Fishback P, Rose J, Snowden KA, Storrs T. New evidence on redlining by federal housing programs in the 1930s. J Urban Econ. 2024;141:103462. doi: 10.1016/j.jue.2022.103462

[10] An B, Orlando AW, Rodnyansky S. The physical legacy of racism: how redlining cemented the modern built environment. Rochester, NY. 2019. doi: 10.2139/ssrn.3500612

[11] Mian A, Rao K, Sufi A. Household balance sheets, consumption, and the economic slump*. Quarterly J Econ. 2013;128(4):1687–726. doi: 10.1093/qje/qjt020

[12] Glaeser EL, Gyourko J. Urban decline and durable housing. J Political Econ. 2005;113(2):345–75. doi: 10.1086/427465

[13] MELZER BT. Mortgage debt overhang: reduced investment by homeowners at risk of default. J Finance. 2017;72(2):575–612. doi: 10.1111/jofi.12482

[14] Haughwout A, Sutherland S, Tracy JS. Negative equity and housing investment. Rochester, NY; 2013.

[15] Gyourko J, Saiz A. Reinvestment in the housing stock: the role of construction costs and the supply side. J Urban Econ. 2004;55(2):238–56. doi: 10.1016/j.jue.2003.09.004

[16] Nicolaides B, Wiese A. Suburbanization in the United States after 1945. Oxford Research Encyclopedia of American History. Available from: https://oxfordre.com/americanhistory/display/10.1093/acrefore/9780199329175.001.0001/acrefore-9780199329175-e-64

[17] Ofulue C. Redlining in Boston: How the Architects of the Past Have Shaped Boston’s Future. The BPR [Internet]. 4 Nov 2021 [cited 17 Sep 2024]. Available from: https://www.bostonpoliticalreview.org/post/redlining-in-boston-how-the-architects-of-the-past-have-shaped-boston-s-future

[18] Nelson RK, Winling L, Marciano R, Connolly N. Mapping inequality. [cited 17 Sep 2024]. Available from: https://dsl.richmond.edu/panorama/redlining/

[19] Leydon. How A Long-Ago Map Created Racial Boundaries That Still Define Boston. GBH [Internet]; 13 Nov 2019 [cited 17 Sep 2024]. Available from: https://www.wgbh.org/news/local/2019-11-12/how-a-long-ago-map-created-racial-boundaries-that-still-define-boston

[20] Miletsky Z, González T. Separatist city: The Mandela, Massachusetts (Roxbury) movement and the politics of incorporation, self-determination, and community control, 1986–1988. Trotter Rev. 2016;23.

[21] Levine H, Harmon L. The death of an American Jewish community: a tragedy of good intentions. New York: Free Press; 1992.

[22] Lima A, Lee J, Kim C. Historical trends in Boston neighborhoods since 1950. Boston Planning and Development Agency Research Division; 2017. Available from: https://www.bostonplans.org/getattachment/89e8d5ee-e7a0-43a7-ab86-7f49a943eccb

[23] Lima A, Lee J, Kim C. Historical Boston in context: neighborhood comparisons by decade 1950 to 2015. Boston Planning and Development Agency Research Division; 2017. Available from: http://www.bostonplans.org/getattachment/8aefd3d3-81ae-4a76-bb9c-9e2d6a3fa8c3

[24] K C M, Oral E, Straif-Bourgeois S, Rung AL, Peters ES. The effect of area deprivation on COVID-19 risk in Louisiana. PLoS One. 2020;15(12):e0243028. doi: 10.1371/journal.pone.0243028 33270701 PMC7714106

[25] Frumkin H. COVID-19, the Built Environment, and Health. Environ Health Perspect. 2021;129(7):75001. doi: 10.1289/EHP8888 34288733 PMC8294798

[26] McClure E, Feinstein L, Cordoba E, Douglas C, Emch M, Robinson W, et al. The legacy of redlining in the effect of foreclosures on Detroit residents’ self-rated health. Health Place. 2019;55:9–19. doi: 10.1016/j.healthplace.2018.10.004 30448354 PMC6345551

[27] Krieger J, Higgins DL. Housing and health: time again for public health action. Am J Public Health. 2002;92(5):758–68. doi: 10.2105/ajph.92.5.758 11988443 PMC1447157

[28] Phipatanakul W, Eggleston PA, Wright EC, Wood RA, National Coooperative Inner-City Asthma Study. Mouse allergen. II. The relationship of mouse allergen exposure to mouse sensitization and asthma morbidity in inner-city children with asthma. J Allergy Clin Immunol. 2000;106(6):1075–80. doi: 10.1067/mai.2000.110795 11112889

[29] Peat JK, Dickerson J, Li J. Effects of damp and mould in the home on respiratory health: a review of the literature. Allergy. 1998;53(2):120–8. doi: 10.1111/j.1398-9995.1998.tb03859.x 9534909

[30] Platt SD, Martin CJ, Hunt SM, Lewis CW. Damp housing, mould growth, and symptomatic health state. BMJ. 1989;298(6689):1673–8. doi: 10.1136/bmj.298.6689.1673 2503174 PMC1836778

[31] Williamson IJ, Martin CJ, McGill G, Monie RD, Fennerty AG. Damp housing and asthma: a case-control study. Thorax. 1997;52(3):229–34. doi: 10.1136/thx.52.3.229 9093337 PMC1758502

[32] Lubin JH, Boice JD Jr. Lung cancer risk from residential radon: meta-analysis of eight epidemiologic studies. J Natl Cancer Inst. 1997;89(1):49–57. doi: 10.1093/jnci/89.1.49 8978406

[33] Lee EK, Donley G, Ciesielski TH, Gill I, Yamoah O, Roche A, et al. Health outcomes in redlined versus non-redlined neighborhoods: A systematic review and meta-analysis. Soc Sci Med. 2022;294:114696. doi: 10.1016/j.socscimed.2021.114696 34995988

[34] Ahmad K, Erqou S, Shah N, Nazir U, Morrison AR, Choudhary G, et al. Association of poor housing conditions with COVID-19 incidence and mortality across US counties. PLoS One. 2020;15(11):e0241327. doi: 10.1371/journal.pone.0241327 33137155 PMC7605696

[35] Hollenbach SJ, Thornburg LL, Glantz JC, Hill E. Associations Between Historically Redlined Districts and Racial Disparities in Current Obstetric Outcomes. JAMA Netw Open. 2021;4(9):e2126707. doi: 10.1001/jamanetworkopen.2021.26707 34591104 PMC8485176

[36] Casillas E, Ye M, Vargo J, Nardone AL, Shete PB, Thakur N. The Color of a Pandemic: The Association Between Historical Residential Redlining and COVID-19 Outcomes in California. In: C14. BURNOUT, DISPARITIES, AND OUTCOMES OF THE COVID-19 PANDEMIC. American Thoracic Society; 2022. p. A3693–A3693. doi: 10.1164/ajrccm-conference.2022.205.1_meetingabstracts.a3693

[37] Choi Y, Unwin J. Racial impact on infections and deaths due to COVID-19 in New York City.

[38] Meier HCS, Mitchell BC. Historic redlining indicator for 2000, 2010, and 2020 US census tracts. Inter-university Consortium for Political and Social Research (ICPSR); 2023.

[39] Boston COVID-19 Dashboard. In: Boston Public Health Commission [Internet]. [cited 17 Sep 2024]. Available from: https://bphc-dashboard.shinyapps.io/BPHC-dashboard/

[40] 2020 Census. Boston Planning & Development Agency; Available from: http://www.bostonplans.org/research/2020-census

[41] Lima A, Kim C, Granberry P. Boston in context: Neighborhoods. 2020 Decennial census redistricting data and 2016-2020 American community survey. Boston Planning & Development Agency Research Division; 2022 Apr. Available from: https://www.bostonplans.org/getattachment/86f801a8-f8a6-4d0c-83ed-b9a63684d6b5

[42] Feigenbaum JJ, Muller C, Wrigley-Field E. Regional and Racial Inequality in Infectious Disease Mortality in U.S. Cities, 1900-1948. Demography. 2019;56(4):1371–88. doi: 10.1007/s13524-019-00789-z 31197611 PMC7258300

[43] Zelner JL, Muller C, Feigenbaum JJ. Racial inequality in the annual risk of Tuberculosis infection in the United States, 1910-1933. Epidemiol Infect. 2017;145(9):1797–804. doi: 10.1017/S0950268817000802 28436340 PMC9203285

[44] Roberts SK. Infectious Fear: Politics, Disease, and the Health Effects of Segregation. University of North Carolina Press; 2009.

[45] Moudon AV, Cook AJ, Ulmer J, Hurvitz PM, Drewnowski A. A neighborhood wealth metric for use in health studies. Am J Prev Med. 2011;41(1):88–97. doi: 10.1016/j.amepre.2011.03.009 21665069 PMC3118096

[46] Stermon M, Lukinbeal C. Institutionalized Racism: Redlined Districts Then and Now in Boston, Detroit, and Los Angeles. Yearbook of the Association of Pacific Coast Geographers. 2021;83(1):81–97. doi: 10.1353/pcg.2021.0007

[47] Arcaya MC, Nidam Y, Binet A, Gibson R, Gavin V. Rising home values and Covid-19 case rates in Massachusetts. Soc Sci Med. 2020;265:113290. doi: 10.1016/j.socscimed.2020.113290 32843186 PMC7420948

[48] Dooling S. Why Some Boston Neighborhoods Have Been Hit Harder By The Pandemic Than Others. In: WBUR [Internet]; 30 Apr 2020 [cited 17 Sep 2024]. Available: https://www.wbur.org/news/2020/04/30/covid-19-coronavirus-boston-neighborhoods

[49] Hortion JO, Horton LE. Park Archives: Boston African American National Historic Site. [cited 17 Sep 2024]. Available: https://npshistory.com/publications/boaf/index.htm

[50] Rigby D, Seguin C. The Racial Position of European Immigrants 1883–1941: Evidence from Lynching in the Midwest. Social Currents. 2018;5(5):438–57. doi: 10.1177/2329496518780921

[51] Bluestone B, Stevenson M, Massagli M, Moss P, Tilly C. Boston renaissance, the: race, space, and economic change in an American metropolis. Russell Sage Foundation; 2000.

[52] McArdle N. Race, Place, and Opportunity: Racial Change and Segregation in the Boston Metropolitan Area: 1990-2000. 2003 [cited 17 Sep 2024]. Available from: https://escholarship.org/uc/item/82t2q8k8

[53] Mehdipanah R, McVay KR, Schulz AJ. Historic Redlining Practices and Contemporary Determinants of Health in the Detroit Metropolitan Area. Am J Public Health. 2023;113(S1):S49–57. doi: 10.2105/AJPH.2022.307162 36696614 PMC9877378

[54] Gioielli R. The Tyranny of the Map: Rethinking Redlining. In: The Metropole [Internet]. 3 Nov 2022 [cited 18 Sep 2024]. Available from: https://themetropole.blog/2022/11/03/the-tyranny-of-the-map-rethinking-redlining/

[55] Abuelezam NN, Michel I, Marshall BD, Galea S. Accounting for historical injustices in mathematical models of infectious disease transmission: An analytic overview. Epidemics. 2023;43:100679. doi: 10.1016/j.epidem.2023.100679 36924757 PMC10330874

[56] Redlining and Present-Day Neighborhood Opportunity in the Boston Area | diversitydatakids.org. [cited 16 Sep 2024]. Available from: https://www.diversitydatakids.org/research-library/data-visualization/redlining-and-present-day-neighborhood-opportunity-boston-area

[57] Samuels-Kalow ME, Dorner S, Cash RE, Dutta S, White B, Ciccolo GE, et al. Neighborhood Disadvantage Measures and COVID-19 Cases in Boston, 2020. Public Health Rep. 2021;136(3):368–74. doi: 10.1177/00333549211002837 33729070 PMC8580391

[58] Blacks account for high share of Boston COVID-19 cases - CommonWealth Beacon. [cited 16 Sep 2024]. Available from: https://commonwealthbeacon.org/health-care/blacks-account-for-high-share-of-boston-covid-19-cases/

[59] Brakefield WS, Olusanya OA, White B, Shaban-Nejad A. Social Determinants and Indicators of COVID-19 Among Marginalized Communities: A Scientific Review and Call to Action for Pandemic Response and Recovery. Disaster Med Public Health Prep. 2022;17:e193. doi: 10.1017/dmp.2022.104 35492024 PMC9237492

[60] Student Housing Trends 2018-2019 Academic Year. City of Boston. Available from: https://www.boston.gov/sites/default/files/embed/b/boston_student_housing_trends_ay_18-19_190509.pdf

