# Supplementary material for: The impact of historical redlining policies on community composition and the COVID-19 pandemic in Boston
Source: PLoS One. 2025 Jun 18;20(6):e0324020. doi: 10.1371/journal.pone.0324020 (PMC12176184; doi:10.1371/journal.pone.0324020)

**S1 FILE.** Supplementary Materials for “The impact of historical redlining policies on community composition and the COVID-19 pandemic in Boston.” This supplement contains an additional table with linear regression results and a redlining map of Historical Redlining Grades.

**Supplementary Table 1**: Linear regression results between ZCTA median home value and ZCTA historical redlining indicator (higher score indicates worse rating)

| Characteristics | Estimate (95% CI) |
| --- | --- |
| Historical redlining indicator | 0.379 (0.25, 0.51) |

**Supplementary Figure 1:** Boston ZCTAs excluded (N = 3) due to lack of Redlining Grade


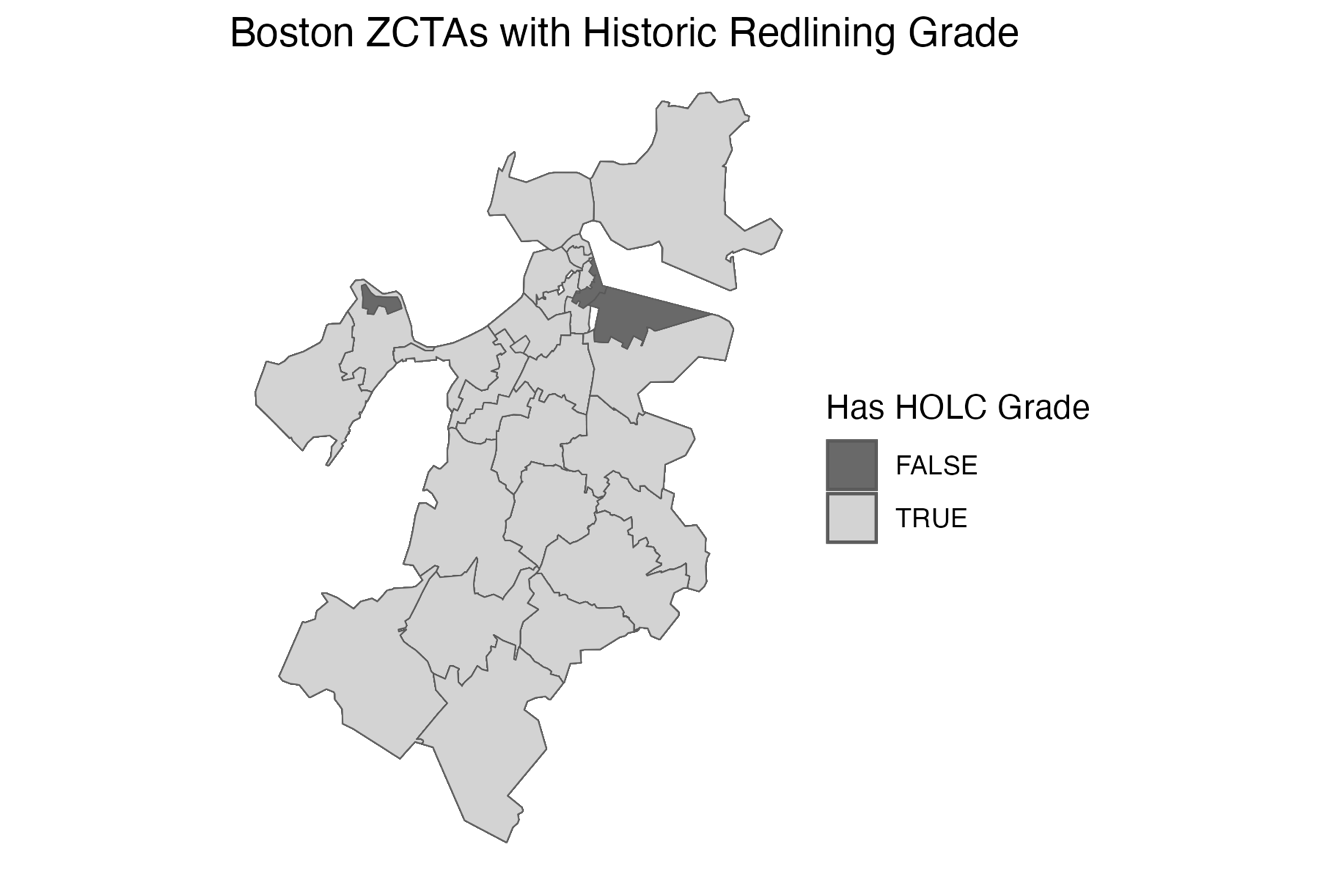

Supplement: S1 File — This supplement contains an additional table with linear regression results and a redlining map of Historical Redlining Grades. (DOCX) [file pone.0324020.s001.docx]
